# Lentivirus-based production of human chimeric antigen receptor macrophages from peripheral blood

**DOI:** 10.1186/s40364-024-00703-9

**Published:** 2025-01-03

**Authors:** Ji U Choi, Yeongrin Kim, Da Yeon Lee, Jin Song Park, Moonjung Jeun, Heung Kyoung Lee, Chi Hoon Park

**Affiliations:** 1 Bio & Drug Discovery Division, Korea Research Institute of Chemical Technology, Daejeon, Republic of Korea; 2 Medicinal Chemistry and Pharmacology, Korea University of Science and Technology, Daejeon, Republic of Korea; 3 College of Pharmacy, Chungnam National University, Daejeon, Republic of Korea

**Keywords:** CAR macrophages, Lentivirus, Human PBMC, Primary human macrophages

## Abstract

Although chimeric antigen receptor (CAR) T cell therapy has shown remarkable efficacy against leukemic cells, it still has critical limitations. CAR macrophage has been regarded as a potential alternative to CAR T cells. However, due to the difficulties in gene transduction into macrophages, the production of primary human CAR macrophages from peripheral blood mononuclear cells (PBMC) using lentivirus is highly challenging. Here, we report on how to generate CAR macrophages from human PBMC with lentiviral particles. Using our lentiviral protocol, we produced functional CAR macrophages to lyse and phagocytose target cancer cells efficiently.

**Supplementary Information:**

The online version contains supplementary material available at 10.1186/s40364-024-00703-9.

**To the Editor**

Although CAR T cell therapy revolutionized the cancer treatment, most of the tumors still remain incurable [1–3]. Macrophage has been considered as an alternative to T cells in CAR-based cell therapy, for they have ability to infiltrate tumors, attack cancer cells orchestrating immune suppressive environments, and induce the attack of T cells against tumor through neo-antigen presentation [4]. However, gene transduction into macrophages using lentiviral systems has been challenging, primarily due to low levels of gene expression and high toxicity [5]. Here, we report how to make functional human primary CAR macrophages from PBMC using lentivirus. Furthermore, we demonstrate their robust lytic and phagocytic activities against target cancer cells.

Human macrophages were isolated from the whole blood donated by healthy volunteers (Fig. 1A, B). We observed that polybrene, a commonly used transduction enhancer, is highly toxic to macrophages (Fig. 1C). Moreover, transduction without polybrene yielded significantly better results (Fig. 1D). Our data also indicate that overnight incubation of lentivirus is more effective for gene transduction into macrophages compared to spinoculation for 1.5 h (Fig. 1E). To further enhance transduction efficiency, we developed lentiviral particles incorporating a codon-optimized VSV-G gene (VSVg-CO) (Fig. 1F). Next, we investigated the optimal timing for gene transduction following monocyte isolation from PBMCs. Macrophages were infected either 3 days or 7 days after monocyte isolation. Morphologically, the isolated cells resembled monocytes at 3 days and macrophages at 7 days post-isolation, respectively. Our results show that later infection (7 days) is significantly more effective for gene transduction (Fig. 1G). Additionally, we found that the EF1α promoter maximized gene expression in macrophages (Fig. 1H). Based on these findings, we generated EF1α-driven, codon-optimized VSVg-pseudotyped lentiviral particles for efficient gene transduction into macrophages.

**Fig. 1:**
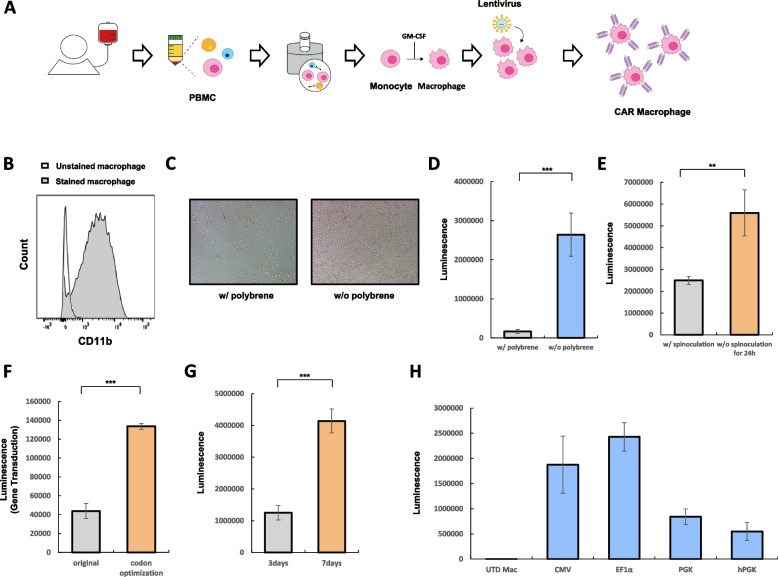
**A** Schematic diagram of CAR macrophage production from human PBMC. **B** FACS analysis of isolated macrophage from PBMC. **C** Macrophages were cultured with or without polybrene. **D** Luciferase gene was transduced by lentivirus with or without polybrene. Luminescence was measured 5 days after gene transduction. **E** Lentiviral luciferase gene was transduced into macrophage with or without spinoculation. Luminescence was measured 5 days after gene transduction. **F** Luciferase gene was transduced into macrophage by original VSVg or codon optimized VSVg pseudotyped lentivirus. Luminescence was measured 5 days after gene transduction. **G** Luciferase gene was transduced into macrophages 3 days or 7 days after macrophage isolation. Luminescence was measured 5 days after gene transduction. **H** Luciferase expressions driven by each promoter in macrophage were investigated through luminescence measurement

Using our protocol, we successfully generated CD19 or cMet targeting CAR macrophages from human PBMCs. The CAR constructs included the CD28 transmembrane and cytosolic domains, as well as the CD3ζ cytosolic domain (Fig. 2). CAR expression was confirmed through western blot and FACS analysis (Fig. 2B, D). Importantly, gene transduction did not alter the morphology of the macrophages, indicating that the process did not cause any damage to the cells (Fig. 2C). Additionally, we assessed the stability of CAR expression over time. As shown in Fig. 2E, CAR expression remained stable for up to 20 days following gene transduction.

**Fig. 2:**
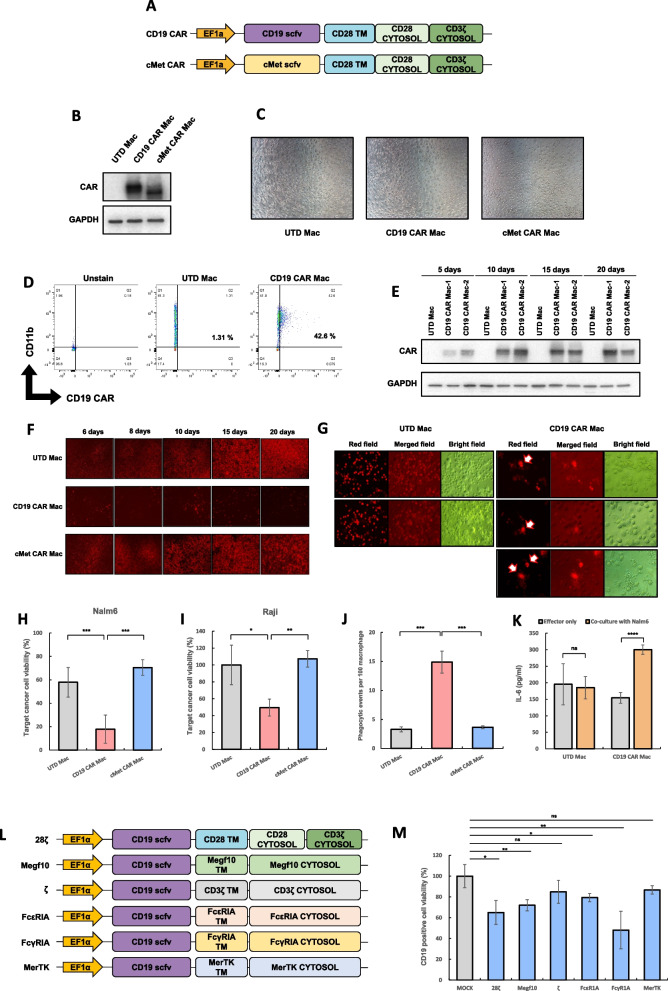
**A** Schematic representation of CAR constructs. **B** CAR expression in each macrophage was detected by western blot. UTD Mac indicates untransduced macrophage. **C** Cell morphology of CAR macrophage was taken 7 days after CAR gene transduction. **D** CAR expression on macrophage surface was measured by FACS 7 days after CAR gene transduction. **E** After gene transduction into macrophage, cell lysates were prepared on 5, 10, 15 or 20 days for western blot. **F** Microscopic images of fluorescence were taken on indicated days after CAR macrophages were incubated with Nalm6-mcherry cells. **G** For phagocytic assay, CAR macrophages were incubated with Nalm6-mcherry cells. Microscopic images were taken 10 days after co-incubation of macrophage and Nalm6. **H**-**I** Luminescence was measured 5 days after CAR macrophages were co-cultured with Nalm6-luciferase cells (H) or Raji-luciferase cells (I). **J** Phagocytic events per 100 macrophages were counted 10 days after CAR macrophages were co-cultured with Nalm6 cells. **K** Secreted IL-6 was measured 7 days after co-culture of CAR macrophages with Nalm6 cells. **L** Constructs of each CD19 CAR genes possessing different transmembrane and cytoplasmic domains. **M** CAR macrophages were produced by transduction of several types of CD19 CAR genes into macrophages. Luminescence was measured 5 days after each CAR macrophages were co-cultured with Nalm6-luciferase cells

We conducted a lysis assay to evaluate the activity of CAR macrophages. CAR macrophages were co-cultured with Nalm6-mcherry cells for several days. As shown in Fig. 2F, Nalm6 cells were specifically lysed by CD19 CAR macrophages. Similarly, a luciferase-based lysis assay demonstrated that CD19 CAR macrophages effectively lysed CD19-positive cancer cells (Fig. 2H). Furthermore, Raji cells, another CD19-positive cancer cell line, were also lysed by CD19 CAR macrophages (Fig. 2I). Next, we performed a phagocytosis assay to assess the hallmark function of macrophages: the engulfment of target cells. Microscopic images revealed red fluorescence signals within CD19 CAR macrophages, indicating that they had phagocytosed Nalm6 cells (Fig. 2G, J). In contrast, red fluorescence signals were rarely observed in UTD macrophages. Additionally, an ELISA assay demonstrated that CAR macrophages secreted IL-6 when incubated with antigen-positive cancer cells (Fig. 2K). These findings indicate that CAR macrophages generated using our lentiviral protocol exhibit potent anti-cancer activities, including efficient lysis and phagocytosis of cancer cells.

Using our lentiviral system, we sought to identify the most effective signaling domain for CAR constructs in CAR macrophages. We tested several signaling domains, including 28ζ, Megf10, ζ, FcεRIA, FcγRIA, and MerTK (Fig. 2L). All CAR constructs consisted of an extracellular CD19 scFv, a transmembrane domain, and an intracellular signaling domain derived from these activation domains (Supplementary Table 1). Our luciferase-based lysis assay revealed that FcγRIA CAR macrophages exhibited the most potent lytic activity against target cancer cells (Fig. 2M).

In this study, we developed a protocol to generate CAR macrophages from human PBMCs using lentiviral transduction. There are many papers describing CAR macrophages. However, many of them studied with mouse macrophages, macrophage cell lines, or macrophages differentiated from human iPSCs [6–10]. Others studied with adenovirus-based primary human CAR macrophage [11, 12]. No paper reported on the functional primary human CAR macrophages derived from peripheral blood through lentiviral transduction.

In conclusion, we present a protocol for generating functional CAR macrophages from PBMCs using lentivirus. Moreover, we demonstrated that lentivirus-based human CAR macrophages effectively destroy cancer cells, highlighting their therapeutic potential.

## Supplementary Information

Supplementary Material 1. 

## Data Availability

No datasets were generated or analysed during the current study.

## Abbreviations

CAR: Chimeric antigen receptor
PBMC: Peripheral blood mononuclear cells
VSVg: Vesicular stomatitis virus G
EF1α: Elongation factor 1-alpha
IL-6: Interleukin 6
GM-CSF: Granulocyte–macrophage colony-stimulating factor
Megf10: Multiple EGF Like Domains 10
FcεRIA: Fc epsilon receptor I A
FcγRIA: Fc gamma receptor I A
MerTK: Mer tyrosine kinase

## References

[1] Dai H, Tong C, Shi D, Chen M, Guo Y, Chen D, Han X, Wang H, Wang Y, Shen P. Efficacy and biomarker analysis of CD133-directed CAR T cells in advanced hepatocellular carcinoma: a single-arm, open-label, phase II trial. Oncoimmunology. 2020;9(1):1846926. 33312759 10.1080/2162402X.2020.1846926 PMC7714531

[2] Fu Q, Zheng Y, Fang W, Zhao Q, Zhao P, Liu L, Zhai Y, Tong Z, Zhang H, Lin M, et al. RUNX-3-expressing CAR T cells targeting glypican-3 in patients with heavily pretreated advanced hepatocellular carcinoma: a phase I trial. EClinicalMedicine. 2023;63: 102175. 37680942 10.1016/j.eclinm.2023.102175 PMC10480529

[3] Papa S, Adami A, Metoudi M, Beatson R, George MS, Achkova D, Williams E, Arif S, Reid F, Elstad M *et al*: Intratumoral pan-ErbB targeted CAR-T for head and neck squamous cell carcinoma: interim analysis of the T4 immunotherapy study. J Immunother Cancer 2023;11(6):e007162. 10.1136/jitc-2023-007162 PMC10277526 37321663

[4] Noy R, Pollard JW. Tumor-associated macrophages: from mechanisms to therapy. Immunity. 2014;41(1):49–61. 25035953 10.1016/j.immuni.2014.06.010 PMC4137410

[5] Sharova N, Wu Y, Zhu X, Stranska R, Kaushik R, Sharkey M, Stevenson M. Primate lentiviral Vpx commandeers DDB1 to counteract a macrophage restriction. PLoS Pathog. 2008;4(5): e1000057. 18451984 10.1371/journal.ppat.1000057 PMC2323106

[6] Chen C, Jing W, Chen Y, Wang G, Abdalla M, Gao L, Han M, Shi C, Li A, Sun P *et al*: Intracavity generation of glioma stem cell-specific CAR macrophages primes locoregional immunity for postoperative glioblastoma therapy. Sci Transl Med 2022;14(656):eabn1128. 10.1126/scitranslmed.abn1128 35921473

[7] Huo Y, Zhang H, Sa L, Zheng W, He Y, Lyu H, Sun M, Zhang L, Shan L, Yang A, et al. M1 polarization enhances the antitumor activity of chimeric antigen receptor macrophages in solid tumors. J Transl Med. 2023;21(1):225. 36978075 10.1186/s12967-023-04061-2 PMC10044396

[8] Lei A, Yu H, Lu S, Lu H, Ding X, Tan T, Zhang H, Zhu M, Tian L, Wang X, et al. A second-generation M1-polarized CAR macrophage with antitumor efficacy. Nat Immunol. 2024;25(1):102–16. 38012418 10.1038/s41590-023-01687-8

[9] Zhang J, Webster S, Duffin B, Bernstein MN, Steill J, Swanson S, Forsberg MH, Bolin J, Brown ME, Majumder A, et al. Generation of anti-GD2 CAR macrophages from human pluripotent stem cells for cancer immunotherapies. Stem cell reports. 2023;18(2):585–96. 36638788 10.1016/j.stemcr.2022.12.012 PMC9968983

[10] Kang M, Lee SH, Kwon M, Byun J, Kim D, Kim C, Koo S, Kwon SP, Moon S, Jung M, et al. Nanocomplex-Mediated In Vivo Programming to Chimeric Antigen Receptor-M1 Macrophages for Cancer Therapy. Adv Mater. 2021;33(43): e2103258. 34510559 10.1002/adma.202103258

[11] Biglari A, Southgate TD, Fairbairn LJ, Gilham DE. Human monocytes expressing a CEA-specific chimeric CD64 receptor specifically target CEA-expressing tumour cells in vitro and in vivo. Gene Ther. 2006;13(7):602–10. 16397508 10.1038/sj.gt.3302706

[12] Klichinsky M, Ruella M, Shestova O, Lu XM, Best A, Zeeman M, Schmierer M, Gabrusiewicz K, Anderson NR, Petty NE, et al. Human chimeric antigen receptor macrophages for cancer immunotherapy. Nat Biotechnol. 2020;38(8):947–53. 32361713 10.1038/s41587-020-0462-y PMC7883632

