# Supplementary material for: Lentivirus-based production of human chimeric antigen receptor macrophages from peripheral blood
Source: Biomark Res. 2025 Jan 3;13:1. doi: 10.1186/s40364-024-00703-9 (PMC11697635; doi:10.1186/s40364-024-00703-9)
Supplement: Supplementary file 1 — Supplementary Material 1. [file 40364_2024_703_MOESM1_ESM.docx]

**Supplementary Information**

**Materials and methods**

Cell lines

Lenti-X 293T cells were maintained in DMEM/high glucose medium (Hyclone, Logan, UT, USA; #SH30243) supplemented with 10 % fetal bovine serum (Gibco, Thermo Fisher Scientific, Dublin, Ireand; #16000-044). Nalm6 and Raji cells were cultured in RPMI 1640 medium (HyClone, Logan, UT, USA; #SH30027) supplemented with 10 % FBS. All the cells were incubated in a humidified incubator at 37°C with 5 % CO2.

Plasmid construction

CAR gene was cloned into lentiviral vector. The PCR product of CMV, EF1α, PGK and hPGK promoters were ligated into luciferase-lentiviral vector. The second-generation CAR construct contained CD19 scFv or cMet scFv, transmembrane domain (CD28, Megf10, CD3ζ, FcεRIA, FcγRIA and MerTK), and cytoplasmic domain (CD28, Megf10, CD3ζ, FcεRIA, FcγRIA and MerTK).

Lentivirus production

On the first day, 8×10^6^ cells Lenti-X 293T cells were seeded on 10 mm tissue culture dishes. A day later, Lenti-X 293T cells were co-transfected with three plasmids, psPAX2 (Addgene, Watertown, MA, USA; #12260), pMD2.G (Addgene; #12259) and transfer plasmid. On the third day, the medium of transfected Lenti-X 293T cells was replaced with fresh DMEM medium with 10 % FBS. Lentivirus supernatant was harvested 24h and 48h after medium change. The lentivirus supernatant was filtered through a 0.45 μm pore sized polyethersulfone (PES) membrane syringe filter (Merck Millipore, Burlington, MA, USA; #SLHP033RB). Viral RNA was isolated from a portion of the lentiviral supernatant using the NucleoSpin RNA Virus kit (MACHEREY-NAGEL, Duren, Germany, #740956.50), and the viral RNA copy number was quantified using the Lenti-X™ qRT-PCR Titration Kit (Takara, Kusatsu, Shiga, Japan, #631235) to determine the titer.

PBMC isolation

Human peripheral blood mononuclear cells (PBMCs) were obtained from healthy donors who provided written informed consent in accordance with protocols approved by Korea National Institute for Bioethics Policy Institutional Review Board (IRB; approval no. P01-201607-31-003). All experimental protocols using PBMCs were approved by IRB. All methods using blood samples were performed in accordance with the institutional biosafety guidelines. PBMCs were isolated by density gradient centrifugation. Whole blood was mixed with 1 X PBS with 2 % FBS at 1:1 ratio. The diluted blood is gently layered on top of the density gradient medium in an equal ratio to lymphoprep (STEMCELL technologies, Vancouver, BC, Canada; #07851). Centrifuge at 800 x g for 20 min with the brake off. The upper layer was removed and the plasma layer was collected. The cells were washed twice by centrifugation at 300 x g for 8 min with 1 X PBS with 2 % FBS. The cells were resuspended in stock buffer (90% FBS and 10% DMSO) and then cryopreserved in liquid nitrogen tanks.

Production of CAR macrophages

Macrophages were isolated from cryopreserved PBMCs using EasySep Human monocyte cell isolation kit (STEMCELL technologies; #19359). Isolated monocytes were differentiated into macrophages with RPMI 1640 medium containing 10 % FBS and 50 ng/ml GM-CSF (Peprotech; #300-03). Macrophages were either co-cultured with lentiviral supernatant (10^7^ viral particles) for 24 hours or transduced via spinoculation with lentiviral supernatant at 1,000×g for 1.5 hours. For gene transduction, 8 µg/mL polybrene was included in the medium. After gene transduction, the lentivirus supernatant was removed and replaced with fresh medium containing GM-CSF. The CAR macrophages were cultured with the addition of GM-CSF to the medium every 3 days. CAR expression was verified by FACS and western blot.

Western blotting

To confirm the expression of CAR protein, the cell lysates were harvested using 1 X sample buffer (10% glycerol, 2% SDS, 50 mM Tris-HCl (pH 6.8), 3% β-mercaptoethanol). Lysates were boiled for 10 min at 100°C and were loaded into 4–15 % gradient Mini-PROTEAN TGX gels (BioRad, Hercules, CA, USA; #456-1086). Electrophoresis was performed at 80 V and the gel transfer was carried out at 250 mA. After being blocked for 1h in blocking buffer (5 % skimmed milk in 1 X TBS-T), membrane was washed with 1 X TBS-T. CAR protein expression was detected by CD3ζ (BD Biosciences, Franklin Lakes, NJ, USA; #551034). GAPDH (Cell Signaling Technology, Danvers, MA, USA; #5174S) was used as loading controls. The membrane, incubated overnight with the primary antibody at 4°C, was washed using 1 X TBS-T. Next, membrane was incubated with a horseradish peroxidase (HRP)-conjugated secondary antibody (Thermo Fisher Scientific; #31430 and 31460) for 1h. Signal intensity was detected using SuperSignal West Pico solution (Thermo Fisher Scientific; #1856135 and 1856136) and SuperSignal West Femto solution (Thermo Fisher Scientific; #1859023 and 1859022). Images were visualized using Sensi-Q2000 Chemidoc (LugenSci, Bucheon, Republic of Korea) instruments.

Flow cytometry analysis

To stain the cell surface, the harvested 1X10^6^ cells/ml were washed twice with cold stain buffer (1 X PBS with 0.2 % BSA and 0.08 % sodium azide). The cells were incubated with 2.5 ug/ml of Human CD19 protein Fc Tag (ACRO Biosystems, Newark, DE, USA; #H5251) or APC anti-human CD11b (BioLegend; #301310)) for 30 min on ice. After washing the cells with cold stain buffer (repeated twice), they were incubated with PE anti-human IgG Fc (BioLegend; #410708) for 30 min on ice. The cells were washed again with cold stain buffer (repeated twice) and then fixed using 4 % paraformaldehyde (Biosesang, Yongin, Republic of Korea; P2031) for 10 min on ice. Flow cytometry analysis were performed using BD FACS Canto II. FACS data were analyzed with Flow Jo (BD Biosciences, Franklin Lakes, NJ, USA).

Cell cytotoxicity assay

The anti-cancer function of CAR macrophages was measured using the Bright-Glo luciferase-based killing Assay System (Promega, Madison, WI, USA; #E2650). Nalm6 and Raji cells were transduced with the luciferase gene. For luciferase-based killing assay, CAR macrophages and Nalm6 or Raji cells were co-cultured in a 96 well plate for 5 days at 37°C with 5 % CO2. Bright-Glo Luciferase assay reagent was added to the effector/target cell mixture. Luminescence was measured using EnVision reader (PerkinElmer, Watertown, MA, USA). Cell viability was calculated as Formula :

$$( \frac{Luminescence \left[ Target cells with Effector cells \right]}{Luminescence \left[ Target cells only \right]} )\times100$$

Microscopy-based phagocytosis assay

To evaluate the phagocytic activity, macrophages were co-cultured with target cells for 6-20 days. Nalm6 cells were engineered to express mcherry protein for red signal. The fluorescence intensity of Nalm6 cells was imaged using Nikon TE-300. Microscopic images were randomly selected.

Cytokine release assay

To measure the level of secreted cytokine, medium from mixture of CAR macrophages and Nalm6 cells were collected. Enzyme-linked immune absorbance assay (ELISA) kits were purchased from BioLegend (430504, San Diego, CA, USA). ELISA assay was performed according to the manufacturer’s protocol.

Statistical analysis

GraphPad Prism version 10.1 (GraphPad Software, San Diego, CA, USA) was used to conduct statistical analyses. Statistical significance was evaluated using unpaired t-test (ns: non-significant, *p<0.05, **p<0.01, ***p<0.001; ****p<0.0001) (two-tailed p-value).

**Supplementary Table**

| **GENES** | **SEQUENCE** |  |
| --- | --- | --- |
| **28ζ** | MLLLVTSLLLCELPHPAFLLIPDIQMTQTTSSLSASLGDRVTISCRASQDISKYLNWYQQKPDGTVKLLIYHTSRLHSGVPSRFSGSGSGTDYSLTISNLEQEDIATYFCQQGNTLPYTFGGGTKLEITGSTSGSGKPGSGEGSTKGEVKLQESGPGLVAPSQSLSVTCTVSGVSLPDYGVSWIRQPPRKGLEWLGVIWGSETTYYNSALKSRLTIIKDNSKSQVFLKMNSLQTDDTAIYYCAKHYYYGGSYAMDYWGQGTSVTVSSAAADYKDDDDKIEVMYPPPYLDNEKSNGTIIHVKGKHLCPSPLFPGPSKPFWVLVVVGGVLACYSLLVTVAFIIFWVRSKRSRLLHSDYMNMTPRRPGPTRKHYQPYAPPRDFAAYRSPRGGMHRVKFSRSADAPAYQQGQNQLYNELNLGRREEYDVLDKRRGRDPEMGGKPRRKNPQEGLYNELQKDKMAEAYSEIGMKGERRRGKGHDGLYQGLSTATKDTYDALHMQALPPR | **Black : FMC63**  **GREEN : FLAG**  **YELLOW:CD28**  **BLUE : CD3ζ** |
| **Megf10** | MLLLVTSLLLCELPHPAFLLIPDIQMTQTTSSLSASLGDRVTISCRASQDISKYLNWYQQKPDGTVKLLIYHTSRLHSGVPSRFSGSGSGTDYSLTISNLEQEDIATYFCQQGNTLPYTFGGGTKLEITGSTSGSGKPGSGEGSTKGEVKLQESGPGLVAPSQSLSVTCTVSGVSLPDYGVSWIRQPPRKGLEWLGVIWGSETTYYNSALKSRLTIIKDNSKSQVFLKMNSLQTDDTAIYYCAKHYYYGGSYAMDYWGQGTSVTVSSAAADYKDDDDKVIIVGNLNSLSRTSTALPADSYQIGAIAGIIILVLVVLFLLALFIIYRHKQKGKESSMPAVTYTPAMRVVNADYTISGTLPHSNGGNANSHYFTNPSYHTLTQCATSPHVNNRDRMTVTKSKNNQLFVNLKNVNPGKRGPVGDCTGTLPADWKHGGYLNELGAFGLDRSYMGKSLKDLGKNSEYNSSNCSLSSSENPYATIKDPPVLIPKSSECGYVEMKSPARRDSPYAEINNSTSANRNVYEVEPTVSVVQGVFSNNGRLSQDPYDLPKNSHIPCHYDLLPVRDSSSSPKQEDSGGSSSNSSSSSE | **Black : FMC63**  **GREEN : FLAG**  **Purple : Megf10** |
| **ζ** | MLLLVTSLLLCELPHPAFLLIPDIQMTQTTSSLSASLGDRVTISCRASQDISKYLNWYQQKPDGTVKLLIYHTSRLHSGVPSRFSGSGSGTDYSLTISNLEQEDIATYFCQQGNTLPYTFGGGTKLEITGSTSGSGKPGSGEGSTKGEVKLQESGPGLVAPSQSLSVTCTVSGVSLPDYGVSWIRQPPRKGLEWLGVIWGSETTYYNSALKSRLTIIKDNSKSQVFLKMNSLQTDDTAIYYCAKHYYYGGSYAMDYWGQGTSVTVSSAAADYKDDDDKQSFGLLDPKLCYLLDGILFIYGVILTALFLRVKFSRSADAPAYQQGQNQLYNELNLGRREEYDVLDKRRGRDPEMGGKPQRRKNPQEGLYNELQKDKMAEAYSEIGMKGERRRGKGHDGLYQGLSTATKDTYDALHMQALPPR | **Black : FMC63**  **GREEN : FLAG**  **Red : ζ** |
| **FcεR1A** | MLLLVTSLLLCELPHPAFLLIPDIQMTQTTSSLSASLGDRVTISCRASQDISKYLNWYQQKPDGTVKLLIYHTSRLHSGVPSRFSGSGSGTDYSLTISNLEQEDIATYFCQQGNTLPYTFGGGTKLEITGSTSGSGKPGSGEGSTKGEVKLQESGPGLVAPSQSLSVTCTVSGVSLPDYGVSWIRQPPRKGLEWLGVIWGSETTYYNSALKSRLTIIKDNSKSQVFLKMNSLQTDDTAIYYCAKHYYYGGSYAMDYWGQGTSVTVSSAAADYKDDDDKWQLDYESEPLNITVIKAPREKYWLQFFIPLLVVILFAVDTGLFISTQQQVTFLLKIKRTRKGFRLLNPHPKPNPKNN | **Black : FMC63**  **GREEN : FLAG**  **Orange : FcεR1A** |
| **FcγR1A** | MLLLVTSLLLCELPHPAFLLIPDIQMTQTTSSLSASLGDRVTISCRASQDISKYLNWYQQKPDGTVKLLIYHTSRLHSGVPSRFSGSGSGTDYSLTISNLEQEDIATYFCQQGNTLPYTFGGGTKLEITGSTSGSGKPGSGEGSTKGEVKLQESGPGLVAPSQSLSVTCTVSGVSLPDYGVSWIRQPPRKGLEWLGVIWGSETTYYNSALKSRLTIIKDNSKSQVFLKMNSLQTDDTAIYYCAKHYYYGGSYAMDYWGQGTSVTVSSAAADYKDDDDKNVLKRSPELELQVLGLQLPTPVWFHVLFYLAVGIMFLVNTVLWVTIRKELKRKKKWDLEISLDSGHEKKVISSLQEDRHLEEELKCQEQKEEQLQEGVHRKEPQGAT | **Black : FMC63**  **GREEN : FLAG**  **Pink : FcγR1A** |
| **MerTK** | MLLLVTSLLLCELPHPAFLLIPDIQMTQTTSSLSASLGDRVTISCRASQDISKYLNWYQQKPDGTVKLLIYHTSRLHSGVPSRFSGSGSGTDYSLTISNLEQEDIATYFCQQGNTLPYTFGGGTKLEITGSTSGSGKPGSGEGSTKGEVKLQESGPGLVAPSQSLSVTCTVSGVSLPDYGVSWIRQPPRKGLEWLGVIWGSETTYYNSALKSRLTIIKDNSKSQVFLKMNSLQTDDTAIYYCAKHYYYGGSYAMDYWGQGTSVTVSSAAADYKDDDDKPAHGWVDYAPSSTPAPGNADPVLIIFGCFCGFILIGLILYISLAIRKRVQETKFGNAFTEEDSELVVNYIAKKSFCRRAIELTLHSLGVSEELQNKLEDVVIDRNLLILGKILGEGEFGSVMEGNLKQEDGTSLKVAVKTMKLDNSSQREIEEFLSEAACMKDFSHPNVIRLLGVCIEMSSQGIPKPMVILPFMKYGDLHTYLLYSRLETGPKHIPLQTLLKFMVDIALGMEYLSNRNFLHRDLAARNCMLRDDMTVCVADFGLSKKIYSGDYYRQGRIAKMPVKWIAIESLADRVYTSKSDVWAFGVTMWEIATRGMTPYPGVQNHEMYDYLLHGHRLKQPEDCLDELYEIMYSCWRTDPLDRPTFSVLRLQLEKLLESLPDVRNQADVIYVNTQLLESSEGLAQGSTLAPLDLNIDPDSIIASCTPRAAISVVTAEVHDSKPHEGRYILNGGSEEWEDLTSAPSAAVTAEKNSVLPGERLVRNGVSWSHSSMLPLGSSLPDELLFADDSSEGSEVLM | **Black : FMC63**  **GREEN : FLAG**  **Sky : MerTK** |
